# Impacts of MicroRNA Gene Polymorphisms on the Susceptibility of Environmental Factors Leading to Carcinogenesis in Oral Cancer

**DOI:** 10.1371/journal.pone.0039777

**Published:** 2012-06-28

**Authors:** Yin-Hung Chu, Shu-Ling Tzeng, Chiao-Wen Lin, Ming-Hsien Chien, Mu-Kuan Chen, Shun-Fa Yang

**Affiliations:** 1 Institute of Medicine, Chung Shan Medical University, Taichung, Taiwan; 2 Institute of Biochemistry and Biotechnology, Chung Shan Medical University, Taichung, Taiwan; 3 Department of Medical Research, Chung Shan Medical University Hospital, Taichung, Taiwan; 4 Wan Fang Hospital, Taipei Medical University, Taipei, Taiwan; 5 Graduate Institute of Clinical Medicine, College of Medicine, Taipei Medical University, Taipei, Taiwan; 6 Department of Otorhinolaryngology-Head and Neck Surgery, Changhua Christian Hospital, Changhua, Taiwan; Geisel School of Medicine at Dartmouth, United States of America

## Abstract

**Background:**

MicroRNAs (miRNAs) have been regarded as a critical factor in targeting oncogenes or tumor suppressor genes in tumorigenesis. The genetic predisposition of miRNAs-signaling pathways related to the development of oral squamous cell carcinoma (OSCC) remains unresolved. This study examined the associations of polymorphisms with four miRNAs with the susceptibility and clinicopathological characteristics of OSCC.

**Methodology/Principal Findings:**

A total of 895 male subjects, including 425 controls and 470 male oral cancer patients, were selected. Polymerase chain reaction-restriction fragment length polymorphism (PCR-RFLP) and real-time PCR were used to analyze miRNA146a, miRNA196, miRNA499 and miRNA149 genetic polymorphisms between the control group and the case group. This study determined that a significant association of miRNA499 with CC genotype, as compared to the subjects with TT genotype, had a higher risk (AOR = 4.52, 95% CI = 1.24–16.48) of OSCC. Moreover, an impact of those four miRNAs gene polymorphism on the susceptibility of betel nut and tobacco consumption leading to oral cancer was also revealed. We found a protective effect between clinical stage development (AOR = 0.58, 95% CI = 0.36–0.94) and the tumor size growth (AOR = 0.47, 95% CI = 0.28–0.79) in younger patients (age<60).

**Conclusions:**

Our results suggest that genetic polymorphism of miRNA499 is associated with oral carcinogenesis, and the interaction of the miRNAs genetic polymorphism and environmental carcinogens is also related to an increased risk of oral cancer in Taiwanese.

## Introduction

MircoRNAs (miRNAs) are small fragment RNAs, which contain approximately 20–22 nucleotides, can target specific mRNAs and negatively regulate their translational efficiency and stability [1]. Previous findings have shown that one miRNA could influence expression of several target genes. According to regulating different target RNAs, miRNAs can participate in cellular processes including proliferation, differentiation, and survival [2]. Altered miRNA expression has been observed in several diseases, especially in cancer [3].

In Taiwan, oral cancer is the fourth most common cancer among men. A rapid increase of incidence of oral cancer occurred in the past few years and crude mortality rate of oral cancer was 10.1 per 100,000 in 2007 and ranked as the sixth cause of cancer death [4]. In the past few years, incidence of oral cancer was particularly high in South Asia. This phenomenon appears especially in populations accustomed to chewing areca (betel) nut [5]. Epidemiological studies have also suggested that the susceptibility of oral cancer is mediated by both environmental carcinogens (including alcohol intake, tobacco consumption, and betel nut chewing) and genetic factors [6]. Increasingly more evidence shows that miRNAs are associated with head and neck/oral cancer [7], and several miRNAs have been shown to be unregulated in head and neck cancer [8]. This relationship has also been determined in laboratory research [9].

Single nucleotide polymorphism (SNP) is a variation in the DNA sequence that occurs when nucleotides (A, T, C or G) change in at least 1% of a certain population. Some epidemiologic evidence shows that miRNA genetic variations are associated with progression to oral cancer [10], [11]. While miRNAs have received considerable attention in recent years, SNPs in miRNA and pre-miRNA sequences have been discovered to be connected to their candidate genes [12]. In dbSNP database, over 400 miRNAs SNPs have been documented. To obtain adequate power for evaluating the potential association, we investigated miRNA146a (rs2910164), miRNA149 (rs2292832), miRNA196 (rs11614913), and miRNA499 (rs3746444), those with minor allele frequencies ≥5% and also located at the pre-miRNA regions in the Chinese populations [13], [14]. In another aspect, these four miRNA SNPs have been reported as important for tumorigenesis due to their targeting on several important genes [15]. Therefore, these four miRNA SNPs were selected in this study. We also considered a unique phenomenon in South Asia, where most oral cancer individuals have a betel nut chewing habit. We combined clinical status and laboratory outcomes to determine the relationship between these SNPs and the susceptibility of oral cancer.

## Results

The statistical analysis of demographic characteristics is shown in Table 1. There were significant differences in the distributions of betel-quid chewing (*p*<0.001), alcohol consumption (*p*<0.001), and tobacco use (*p*<0.001) between healthy control subjects and OSCC patients. Because these differences between the two groups may be confounders, we adjusted these characteristics in further statistical analysis.

**Table 1:** Demographical characteristics in 425 healthy controls and 470 patients with oral cancer.

Characteristic	Control	Oral cancer	Total	*p* value
	N = 425 (%)	N = 470 (%)	N = 895 (%)	
Age				
≤60	307 (72.21)	332 (70.64)	639 (71.40)	0.279
>60	118 (27.79)	138 (29.36)	256 (28.60)	
Smoking status				
Yes	202 (47.53)	409 (87.02)	611 (68.27)	<0.001
No	223 (52.47)	61 (12.98)	284 (31.73)	
Alcohol intake				
Yes	185 (43.53)	295 (62.77)	480 (53.63)	<0.001
No	240 (56.47)	175 (37.23)	415 (46.37)	
Betel nut chewing				
Yes	90 (21.18)	371 (78.94)	461 (51.51)	<0.001
No	335 (78.82)	99 (21.06)	434 (48.49)	

The distribution of miRNA SNP genotypes are described in Table 2. In our healthy controls, miRNA polymorphisms (rs2910164, rs11614913, and rs3746444) conformed to the Hardy-Weinberg equilibrium, except for rs2292832 (*p*<0.001). We used logistic regression model to estimate the adjusted odds ratios (AORs). After adjusting age, smoking status, alcohol intake and betel nut chewing habits, we found that miRNA499 CC genotypes exhibited significantly (*p*<0.05) higher risks of 4.52−(95% CI = 1.24–16.48) of having OSCC compared to the corresponding wild-type (WT) homozygotes. However, there was no significantly higher oral cancer risk for individuals with the miRNA146a, miRNA149 and miRNA196 polymorphic gene compared to those with the WT gene.

**Table 2:** Distribution of miRNA genotypes in healthy controls and oral cancer patients.

Gene	Control	Case	OR	AOR^a^
	N = 425 (%)	N = 470 (%)	(95% CI)	(95% CI)
miRNA146ars2910164				
CC	175 (41.18)	174 (37.02)	Reference	Reference
CG	196 (46.12)	242 (51.49)	1.24 (0.94–1.65)	1.18 (0.82–1.70)
GG	54 (12.70)	54 (11.49)	1.01 (0.66–1.55)	0.59 (0.32–1.08)
C allele	546 (64.24)	590 (62.77)	Reference	Reference
G allele	304 (35.76)	350 (37.23)	1.07 (0.88–1.29)	0.91 (0.71–1.17)
miRNA149rs2292832				
TT	315 (74.23)	345 (73.40)	Reference	Reference
CT	84 (19.75)	88 (18.72)	0.96 (0.68–1.34)	0.76 (0.49–1.17)
CC	26 (6.12)	37 (7.88)	1.30 (0.77–2.20)	1.45 (0.75–2.83)
T allele	714 (84.00)	778 (82.77)	Reference	Reference
C allele	136 (16.00)	162 (17.23)	1.09 (0.85–1.40)	1.02 (0.74–1.41)
miRNA196rs11614913				
TT	132 (31.06)	136 (28.94)	Reference	Reference
CT	206 (48.47)	277 (58.94)	1.31 (0.97–1.76)	1.14 (0.78–1.68)
CC	87 (20.47)	57 (12.12)	0.64 (0.42–0.96)*	0.74 (0.42–1.30)
T allele	470 (55.29)	549 (58.40)	Reference	Reference
C allele	380 (44.71)	391 (41.60)	0.88 (0.73–1.06)	0.92 (0.72–1.17)
miRNA499rs3746444				
TT	356 (83.76)	339 (72.13)	Reference	Reference
CT	66 (15.53)	119 (25.32)	1.89 (1.35–2.65)*	1.79 (1.16–2.75)*
CC	3 (0.71)	12 (2.55)	4.20 (1.18–15.02)*	4.52 (1.24–16.48)*
T allele	778 (91.53)	797 (84.79)	Reference	Reference
C allele	72 (8.47)	143 (15.21)	1.94 (1.44–2.62)*	1.81 (1.23–2.65)*

a .AOR adjusted, age, smoking status, alcohol intake and betel nut chewing.

* *p*<0.05.

Considering that risk factors such as betel nut chewing may modify the genetic susceptibility to oral cancer, and the interactive effects between environmental risk factors and genetic polymorphisms of miRNAs are shown in Table 3, Table S1 and Table S2. Subjects with at least one C allele of miRNA499 and a betel nut-chewing habit had respective higher risks of 17.33−(95% CI = 9.63–31.17) of having oral cancer (Table 3). Subjects with at least one G allele of miRNA146a, the C allele of miRNA149, or the C allele of miRNA196, and a betel nut-chewing habit had respective higher risks of 9.93−(95% CI = 6.01–16.41), 8.67−(95% CI = 5.06–14.89), and 10.98−(95% CI = 6.21–19.39) of having oral cancer (Table S1). Similarly, tobacco consumption significantly elevated the oral cancer risk in subjects polymorphic for miRNA146a, miRNA149, miRNA196 and miRNA499 compared to individuals with the WT gene but without smoking (Table 3 and Table S2). We further evaluated the gene-environment statistical interaction between the miRNA polymorphisms, smoking and betel quid on oral cancer (Table 3, Table S1 and Table S2). Statistical significance was found for the interaction between all miRNA polymorphisms, smoking and betel quid on oral cancer development (p<0.05). The above results suggest that miRNAs gene polymorphisms have a strong impact on oral cancer susceptibility in betel nut and/or smoking consumers.

**Table 3:** Association of miRNA 499 genotype and betel nut chewing and smoking status.

Variable	Control	Case	OR	AOR^a^
	N = 425 (%)	N = 470 (%)	(95% CI)	(95% CI)
TT and non-chewing	283 (66.59)	75 (15.96)	Reference	Reference
CT or CC or consumer	125 (29.41)	288 (61.28)	8.69 (6.25–12.09)*	5.95 (4.20–8.44)*
CT or CC with betel nut chewing	17 (4.00)	107 (22.77)	23.75 (13.41–42.06)*	17.33 (9.63–31.17)*
Test for interaction χ^2^ = 31.28 (1 d.f.), *p*<0.001*				
**Variable**	**Control**	**Case**	**OR**	**AOR** b
	**N = 425 (%)**	**N = 470 (%)**	**(95% CI)**	**(95% CI)**
TT and non-smoker	185 (43.53)	40 (8.51)	Reference	Reference
CT or CC or smoker	209 (49.18)	320 (68.09)	7.08 (4.83–10.39)*	3.03 (1.95–4.70)*
CT or CC with smoking	31 (7.29)	110 (23.40)	16.41 (9.71–27.74)*	6.31 (3.42–11.65)*
Test for interaction χ^2^ = 5.30 (1 d.f.), *p* = 0.02*				

a .AOR adjusted, age, smoking status and alcohol intake.

b .AOR adjusted, age, alcohol intake and betel nut chewing.

* *p*<0.05.

To explore the impacts of polymorphic genotypes of miRNAs on the clinical status of OSCC, we further classified OSCC patients into two subgroups: one subgroup with at least one polymorphic allele, and the other subgroup with homozygous WT alleles. Data of the statistical analysis showed that who have polymorphic miRNA499 gene had a protective effect of the tumor size growth (AOR = 0.46, 95% CI = 0.29–0.72) as compared to the patients with wild type (Table 4). However, no significant association between miRNA416a, miRNA149 and miRNA196 gene polymorphisms and the clinicopathologic covariates were observed (data not shown). Moreover, compared to the WT genotype (T/T), patients with at least one polymorphic C allele of miRNA499 showed a protective effect between clinical stage development (AOR = 0.58, 95% CI = 0.36–0.94) and the tumor size growth (AOR = 0.47, 95% CI = 0.28–0.79), in younger patients (age≤60), as shown in Table S3. However, no significant association between miRNA499 gene polymorphisms and the clinicopathologic covariates were observed in elderly patients (age>60) (Table S4).

**Table 4:** Relationship of clinical status and miRNA499 genotypes in oral cancer patients.

Gene	TT	CT/CC	OR	AOR^a^
	N = 339	N = 131	(95% CI)	(95% CI)
Clinical Stage				
Stage I+II	143 (42.18)	69 (52.67)	Reference	Reference
Stage III+IV	196 (57.82)	62 (47.33)	0.66(0.44–0.98)*	0.66 (0.44–1.00)
Tumor Size				
T1+T2	195 (57.23)	98 (74.81)	Reference	Reference
T3+T4	145 (42.77)	33 (25.19)	0.45(0.29–0.71)*	0.46 (0.29–0.72)*
Lymph node metastasis				
Negative	215 (63.42)	88 (67.18)	Reference	Reference
Positive	124 (36.58)	43 (32.82)	0.85 (0.55–1.30)	0.86 (0.56–1.32)
Cell differentiation				
Well differentiated	46 (13.57)	20 (15.27)	Reference	Reference
Moderately or poorly differentiated	293 (86.43)	110 (84.73)	0.87 (0.49–1.54)	0.87 (0.49–1.54)

a .AOR adjusted, age, smoking status, alcohol intake and betel nut chewing.

* *p*<0.05.

## Discussion

In this study, we provided novel information of SNPs of miRNA499 on the association of the oral cancer susceptibility. After combining the major risk factor of oral cancer, these genetic affects can also increase the susceptibility of oral cancer. Considering previous findings, miRNA499 has been determined to exhibit high expression in heart and skeletal muscle tissues [16]. Particularly in the heart, previous studies have also found that high expression of miRNA499 increases the risk of cardiomyocyte hypertrophy and cardiomyopathy. Furthermore, some evidence suggests that miRNA499 can regulate mitochondrial dynamics through by targeting specific proteins such as p53 [17]. Previous studies have also shown that miRNA499 is highly associated with heart diseases by regulate cellular differentiation and proliferation [18], [19]. These findings show that miRNA499 is possibly associated with heart diseases and also carcinogenesis.

Based on the clinical status, previous studies have found that miRNA-486, miRNA-30d, miRNA-1, and miRNA-499 exhibited high expression in serum and were also associated with survival in non-small-cell lung cancer [20]. Other studies have found that the genetic variant in pre-miRNA may contribute to the process of carcinogenesis in breast cancer [14], [21], gastric cancer [22], cervical cancer [23], [24], and head and neck cancer [15]. In this study, we first demonstrated that polymorphism of miRNA499 is associated with oral cancer.

Previous studies have found that miRNA146a may contribute to the tumor progression, metastasis, prognosis, and survival in gastric cancer and oral cancer [25], [26]. Another finding also showed that miRNA146a polymorphism can regulate miRNA146a expression [27]. Several laboratory studies have also discovered that miRNA146a could inhibit cell differentiation and survival in the hematopoietic system [28]. Moreover, connected to cancer, miRNA146a could suppress cell invasion in pancreatic cancer [29]. In detail, some evidence connects miRNA146a with transcription factors such as NF-kB [30]. These findings provide a new vision that miRNA146a could affect cancer progression by regulating cell differentiation but may not affect apoptosis. Although we found no association between miRNA146a and several clinical statuses in our study, based on these findings, future studies could consider joint prognosis status or even survival rate.

Recent studies have identified that miRNA196 may interact with several transcription factors and involve in cancer development and progression [31], [32]. Some findings suggest that overexpression of miRNA196 leads to more favorable prognosis and survival in leukemia [33]. In addition, miRNA196 is associated with inflammation in specific cancers [34]. Furthermore, Christensen et al., reports a polymorphism in the mature sequence of miRNA196a2 in a case-control study (n = 1,039) of head and neck squamous cell carcinoma (HNSCC) [35]. When the authors stratified on tumor site they did not observe a significant association between oral cancer and miRNA196a2, though the effect estimate was protective, similar to the results presented in this study. Although the reason for those discrepancies is not well-known, the different results from the report and the present study may relate to the racial/ethnic difference.

Considering that the causes of carcinomas are complex, we analyzed several risk factors, such as smoking status and betel nut chewing habits, in this study. We expect that these gene polymorphisms could influence the susceptibility of oral cancer. Based on the results, we observed that these polymorphisms significantly increased the odds ratio in each group, possibly because the functions of these miRNAs regulate the differentiations and proliferations in tumor progression. We also found that genetic polymorphism of miRNA499 is associated with distal metastasis of OSCC. A previous study for colorectal cancer has found that overexpression of miRNA499 may facilitate the migration and invasion of cancer cells in vitro, as well as the metastasis to lung and liver in vivo [36]. Additionally, this study also identified forkhead box O4 (FOXO4) and programmed cell death 4 (PDCD4) as direct and functional targets of miRNA499 [36]. Moreover, Reis et al., also reports that PDCD4 as a suppressor of migration and invasion and may be a clinically relevant biomarker with prognostic value in OSCC [37]. These abovementioned findings may provide a preliminary explanation with our finding for the association between miRNA499 and distal metastasis of OSCC. Detailed relevant mechanisms may warrant further studies.

One of the limitations of our study is that information on alcohol, betel nut, and tobacco use is dichotomized into “ever-user” versus “never-user.” As the result, more detailed analysis based on amount, length, and past history of betel nut, alcohol, and tobacco consumption were not able to be performed. Data collection relied on self-reports, for which some individuals may be reluctant to report their habitual use of such substances. Hence, there may be residual confounding effect from betel nut, alcohol, and tobacco use misclassification. Furthermore, the functional role of miRNA in growth or metastasis of oral cancer is worth for further investigation, which will be included in our future work. Clones containing various genotypes of miRNA499 SNPs will be constructed to elucidate the possible functions of miRNA499 (proliferation, cell cycle regulation, migration and invasion) in oral cancer cell lines, as well as the underlying mechanisms. Furthermore, this study revealed the nonconformity of miRNA 149 (rs2292832) genotypes to Hardy-Weinberg equilibrium in the control group. A previous study with 107000 genotypes generated from 443 SNPs has found that genotype distributions for 36 out of 313 assays (11.5%) were deviated from HWE (P<0.05) [38]. Upon searching for the possible reasons, assays for SNPs proved nonspecific or genotyping errors have been identified. However, they also found the deviation from HWE for the remaining 10 SNPs was unexplainable. Although the reason for the nonconformity of miRNA 149 genotypes to HWE in our control group is not explored yet, results from the abovementioned study may provide some direction for our future study.

In conclusion, we discovered a significant association between miRNA499 gene polymorphisms and the susceptibility of oral cancer. However, there are no connections between the SNPs and clinical status. After considering betel nut chewing, which is the most influential factor of oral cancer, we found that those four miRNAs which carrying the mutation genotype may increase the susceptibility of oral cancer. These gene mutations may affect miRNA target efficiency and stability and increase the incidence of oral cancer, but the detailed mechanisms from gene levels to protein levels that ultimately affect tumor development should be classified, perhaps charting a new direction for target therapy.

## Materials and Methods

### Subjects and Specimen Collection

We recruited 470 male patients at Chung Shan Medical University Hospital in Taichung and Changhua Christian Hospital and Show Chwan Memorial Hospital in Changhua, Taiwan as a case group between 2007 and 2011. Meanwhile, controls were enrolled from the physical examination during those three hospitals, which are also the facilities that cases were collected from. At the end of recruitment, a total of 425 male participants that had neither self reported history of cancer of any sites were included. In addition, subjects with oral precancerous disease such as oral submucous fibrosis, leukoplakia, erythroplakia, verrucous hyperplasia, etc. were excluded from control group. The participation rate was approximately 91% for cases and 76% for controls. Since all cases and controls were consecutively collected without any selection, and based on information provided on questionnaires, no significant genetic relationship or family history were found.

As for cases and controls, exposure information, including betel quid chewing (ever- vs. never-user), tobacco use (smoker vs. non-smoker) and alcohol consumption (current heavy drinker, defined by CDC as consuming an average of more than 2 drinks per day vs. not current heavy drinker), were all obtained from questionnaires. While medical information of cases, including TMN clinical staging, primary tumor size, lymph node involvement and histologic grade, were obtained from medical records. Oral cancer patients were staged clinically at the time of diagnosis according to the TNM staging system of the American Joint Committee on Cancer (AJCC) [39]. Tumor differentiation examined by pathologist according to AJCC classification. This study has been reviewed and approved by Institutional Review Board and informed written consent was obtained from each individual.

### DNA Extraction

The whole blood samples, collected from healthy controls and oral cancer patients, were placed into tubes containing EDTA, after centrifuged and stored at −80°C. Venous blood from each subject was drawn into vacutainer tubes containing EDTA and stored at 4°C. Genomic DNA was extracted by QIAamp DNA blood mini kits (Qiagen, Valencia, USA) according to the manufacture’s instructions. DNA was dissolved in TE buffer [10 mM Tris (PH 7.8), 1 mM EDTA] and then quantitated by a measurement of OD260. Final preparation was stored at −20°C and used as templates for polymerase chain reaction.

### Polymerase Chain Reaction-restriction Fragment Length Polymorphism (PCR-RFLP)

**Figure 1:**
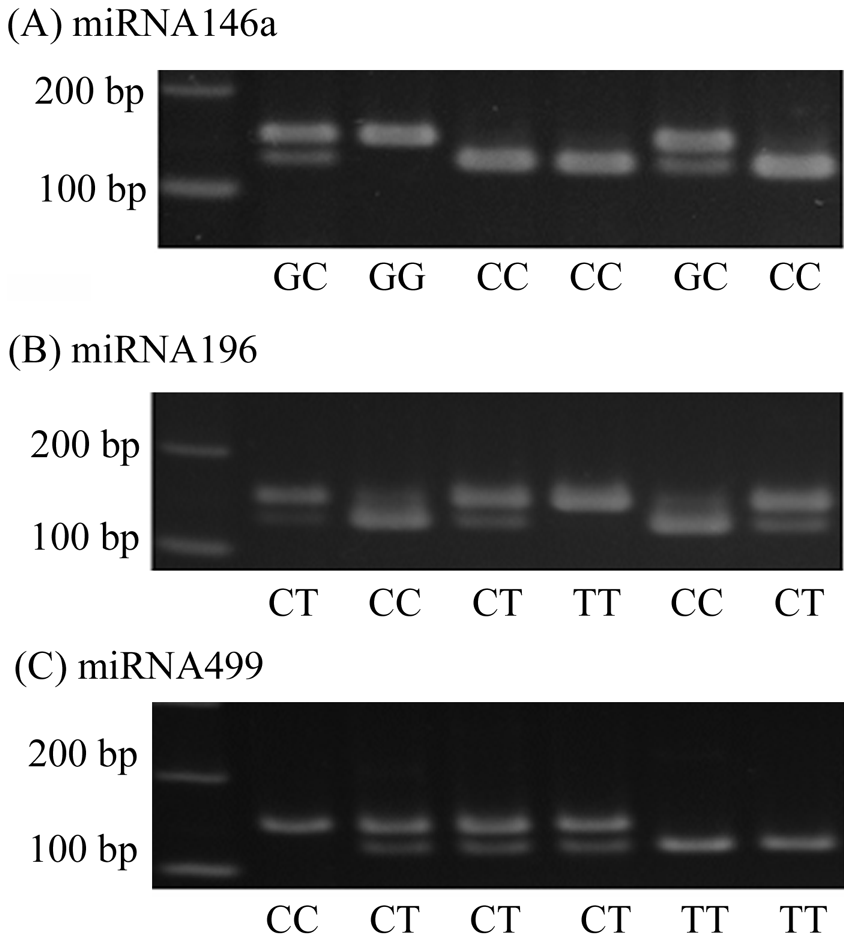
Polymerase chain reaction-restriction fragment length polymorphism of miRNA146a (rs2910164), miRNA196 (rs11614913), miRNA499 (rs3746444) gene. (A) For miRNA146a rs2910164 gene polymorphism, the wild type homozygous alleles (C/C) yielded a 25 and 122-bp products, the heterozygous alleles (C/G) yielded 25-, 122- and 147-bp products, while the mutated type homozygous alleles (G/G) yielded a 147-bp product. (B) For miRNA196 (rs11614913) gene polymorphism, the wild type homozygous alleles (T/T) yielded a 149-bp product, the heterozygous alleles (C/T) yielded 24-, 125- and 149-bp products, while the mutated type homozygous alleles (C/C) yielded a 24- and 125-bp products (C) For miRNA499 (rs3746444) gene polymorphism, the wild type (T/T) yielded 26- and 120-bp products; the heterozygous alleles (C/T) yielded 26-, 120- and 146-bp products, while the mutated type homozygous alleles (C/C) yielded a 146-bp product.

The miRNAs gene rs2910164, rs11614913, and rs3746444 polymorphisms were determined by PCR-RFLP assay [15]. The primers sequences and the restriction enzyme for analysis of those miRNAs gene polymorphisms were described in Table S5. PCR was performed in total 10 µl volume containing 100 ng DNA template, 1.0 µl of 10× PCR buffer (Invitrogen, Carslbad, CA, USA), 0.25 U of Taq DNA polymerase (Invitrogen), 0.2 mM dNTPs (Promega, Madison, WI, USA) and 200 nM of each primer (MDBio Inc. Taipei, Taiwan). The PCR cycling conditions were 5 min at 94°C followed by 35 cycles of 1 min at 94°C, 1 minat 58°C for miRNA146a, 1 min at 63°C for miRNA196a2, and 1 min at 67°C for miRNA499, and 2 min at 72°C, with a final step at 72°C for 20 min to allow a complete extension of all PCR fragments. The results were shown in Figure 1. For each assay, appropriate controls (nontemplate and known genotype) were included in each typing run to monitor reagent contamination. To validate results from PCR-RFLP and for quality control, around 10% of assays were repeated from different batches and several cases of each genotype were confirmed by the DNA sequence analysis.

### Real-time PCR

The allelic discrimination of the miRNA149 rs2292832 gene polymorphisms was assessed with the ABI StepOne™ Real-Time PCR System (Applied Biosystems) and analyzed using SDS v3.0 software (Applied Biosystems), using the TaqMan assay. The final volume for each reaction was 5 µL, containing 2.5 µL TaqMan Genotyping Master Mix, 0.125 µL TaqMan probes mix, and 10 ng genomic DNA. The real-time PCR reaction included an initial denaturation step at 95°C for 10 min, followed by 40 cycles, each consisting of 95°C for 15 sec and 60°C for 1 min.

### Statistical Analysis

The distributions of demographic characteristics and genotype frequencies between cases and controls as well as clinicopathological features in different genotypes were analyzed by Fisher’s exact test, since the small sample size was present in some categories of variables. The odds ratios (ORs) with their 95% confidence intervals (CIs) of the association between genotype frequencies and oral cancer were estimated by multiple logistic regression models, after controlling for covariates. Nonparametric method was used due to not normal distribution of some estimated variables. We fitted a logistic regression model with main effects (miRNAs genetic polymorphism, smoking status and betel quid chewing status), as well as an interaction term between them (miRNAs genotypes*demographic characteristic), comparing the model against a model with only the main effects. Interaction effect was defined as the difference of their deviance, and further assessed using the likelihood ratio test to calculate χ^2^ and *p* values [40]. A *p* value of less than 0.05 was considered significant. The data were analyzed on R statistical software.

## Supporting Information

Table S1 **Association of miRNA genotype and betel nut chewing status.**
(DOC)

Table S2 **Association of miRNA genotype and smoking status.**
(DOC)

Table S3 **Relationship of clinical status and miRNA499 genotypes in oral cancer patients (≤60 only, N = 332).**
(DOC)

Table S4 **Relationship of clinical status and miRNA499 genotypes in oral cancer patients (>60 only, N = 138).**
(DOC)

Table S5 **Primer sequences and PCR conditions for amplification of miRNA SNPs.**
(DOC)
