# Supplementary material for: Impacts of MicroRNA Gene Polymorphisms on the Susceptibility of Environmental Factors Leading to Carcinogenesis in Oral Cancer
Source: PLoS One. 2012 Jun 28;7(6):e39777. doi: 10.1371/journal.pone.0039777 (PMC3386241; doi:10.1371/journal.pone.0039777)
Supplement: Table S1 — Association of miRNA genotype and betel nut chewing status. (DOC) [file pone.0039777.s001.doc]

| Table S1. Association of miRNA genotype and betel nut chewing status. | | | | |
| --- | --- | --- | --- | --- |
| Variable | Control | Case | OR | AOR^a^ |
|  | N=425 (%) | N=470 (%) | (95% CI) | (95% CI) |
| miRNA146a  rs2910164 |  |  |  |  |
| CC and non-chewing | 144 (33.88) | 36 (7.66) | Reference | Reference |
| CG or GG or consumer | 222 (52.24) | 201 (42.77) | 3.62 (2.40-5.47)* | 3.27 (2.12-5.04)* |
| CG or GG with betel nut chewing | 59 (13.88) | 233 (49.57) | 15.80 (9.94-25.12)* | 9.93 (6.01-16.41)* |
| Test for interaction χ^2^ = 80.01 (1 d.f.), *p*<0.001* | | | | |
|  |  |  |  |  |
| miRNA149  rs2292832 |  |  |  |  |
| TT and non-chewing | 251 (59.06) | 74 (15.74) | Reference | Reference |
| CT or CC or consumer | 148 (34.82) | 296 (62.98) | 6.78 (4.90-9.40)* | 4.83 (3.42-6.82)* |
| CT or CC with betel nut chewing | 26 (6.12) | 100 (21.28) | 13.05 (7.89-21.58)* | 8.67 (5.06-14.89)* |
| Test for interaction χ^2^ = 35.52 (1 d.f.), *p*<0.001* | | | | |
|  |  |  |  |  |
| miRNA196  rs11614913 |  |  |  |  |
| TT and non-chewing | 111 (26.12) | 22 (4.68) | Reference | Reference |
| CT or CC or consumer | 245 (57.65) | 191 (40.64) | 3.93 (2.40-6.45)* | 3.25 (1.94-5.46)* |
| CT or CC with betel nut chewing | 69 (16.24) | 257 (54.68) | 18.79 (11.07-31.89)* | 10.98 (6.21-19.39)* |
| Test for interaction χ^2^ = 93.09 (1 d.f.), *p*<0.001* | | | | |
|  | | | | |

a. AOR adjusted, age, smoking status and alcohol intake.

* *p*<0.05
