# Supplementary material for: Impacts of MicroRNA Gene Polymorphisms on the Susceptibility of Environmental Factors Leading to Carcinogenesis in Oral Cancer
Source: PLoS One. 2012 Jun 28;7(6):e39777. doi: 10.1371/journal.pone.0039777 (PMC3386241; doi:10.1371/journal.pone.0039777)
Supplement: Table S2 — Association of miRNA genotype and smoking status. (DOC) [file pone.0039777.s002.doc]

| Table S2. Association of miRNA genotype and smoking status. | | | | |
| --- | --- | --- | --- | --- |
| Variable | Control | Case | OR | AOR^a^ |
|  | N=425 (%) | N=470 (%) | (95% CI) | (95% CI) |
| miRNA146a  rs2910164 |  |  |  |  |
| CC and non-smoker | 93 (21.88) | 28 (5.96) | Reference | Reference |
| CG or GG or smoker | 212 (49.88) | 179 (38.09) | 2.80 (1.76-4.48)* | 1.78 (1.03-3.08)* |
| CG or GG with smoking | 120 (28.24) | 263 (55.96) | 7.28 (4.53-11.70)* | 2.81 (1.62-4.87)* |
| Test for interaction χ^2^ = 16.47 (1 d.f.), *p*<0.001* | | | | |
|  |  |  |  |  |
| miRNA149  rs2292832 |  |  |  |  |
| TT and non-smoker | 165 (38.82) | 50 (10.64) | Reference | Reference |
| CT or CC or smoker | 208 (48.94) | 306 (65.11) | 4.86 (3.38-6.97)* | 2.03 (1.32-3.12)* |
| CT or CC with smoking | 52 (12.24) | 114 (24.26) | 7.24 (4.59-11.41)* | 3.46 (2.00-6.02)* |
| Test for interaction χ^2^ = 11.61 (1 d.f.), *p*<0.001* | | | | |
|  |  |  |  |  |
| miRNA196  rs11614913 |  |  |  |  |
| TT and non-smoker | 79 (18.59) | 16 (3.40) | Reference | Reference |
| CT or CC or smoker | 197 (46.35) | 165 (35.11) | 4.13 (2.33-7.35)* | 2.79 (1.43-5.44)* |
| CT or CC with smoking | 149 (35.06) | 289 (61.49) | 9.58 (5.40-16.98)* | 3.66 (1.93-6.96)* |
| Test for interaction χ^2^ = 11.56 (1 d.f.), *p*<0.001* | | | | |
|  | | | | |

1. AOR adjusted, age, alcohol intake and betel nut chewing.

* *p*<0.05
