# Supplementary material for: Impacts of MicroRNA Gene Polymorphisms on the Susceptibility of Environmental Factors Leading to Carcinogenesis in Oral Cancer
Source: PLoS One. 2012 Jun 28;7(6):e39777. doi: 10.1371/journal.pone.0039777 (PMC3386241; doi:10.1371/journal.pone.0039777)
Supplement: Table S3 — Relationship of clinical status and miRNA499 genotypes in oral cancer patients (≤60 only, N = 332). (DOC) [file pone.0039777.s003.doc]

| Table S3. Relationship of clinical status and miRNA499 genotypes in oral cancer patients (<60 only, N=332) | | | | |
| --- | --- | --- | --- | --- |
| Gene | TT | CT/CC | OR | AOR^a^ |
|  | N=232 | N=100 | (95% CI) | (95% CI) |
| Clinical Stage |  |  |  |  |
| Stage I+ II | 94 (40.52) | 54 (54.00) | Reference | Reference |
| Stage III+IV | 138 (59.48) | 46 (46.00) | 0.58 (0.36-0.93)* | 0.58 (0.36-0.94)* |
|  |  |  |  |  |
| Tumor Size |  |  |  |  |
| T1 + T2 | 134 (57.76) | 75 (75.00) | Reference | Reference |
| T3 + T4 | 98 (42.24) | 25 (25.00) | 0.46 (0.27-0.77)* | 0.47 (0.28-0.79)* |
|  |  |  |  |  |
| Lymph node metastasis |  |  |  |  |
| Negative | 144 (62.07) | 69 (69.00) | Reference | Reference |
| Positive | 88 (37.93) | 31 (31.00) | 0.74 (0.45-1.21) | 0.72 (0.43-1.19) |
|  |  |  |  |  |
| Cell differentiation |  |  |  |  |
| Well differentiated | 31 (13.36) | 16 (16.00) | Reference | Reference |
| Moderately or poorly differentiated | 201 (86.64) | 84 (84.00) | 0.81 (0.42-1.56) | 0.80 (0.42-1.55) |

a. AOR adjusted, age, smoking status, alcohol intake and betel nut chewing.

* *p*<0.05
