# Supplementary material for: Impacts of MicroRNA Gene Polymorphisms on the Susceptibility of Environmental Factors Leading to Carcinogenesis in Oral Cancer
Source: PLoS One. 2012 Jun 28;7(6):e39777. doi: 10.1371/journal.pone.0039777 (PMC3386241; doi:10.1371/journal.pone.0039777)
Supplement: Table S4 — Relationship of clinical status and miRNA499 genotypes in oral cancer patients (>60 only, N = 138). (DOC) [file pone.0039777.s004.doc]

| Table S4. Relationship of clinical status and miRNA499 genotypes in oral cancer patients (>60 only, N=138) | | | | |
| --- | --- | --- | --- | --- |
| Gene | TT | CT/CC | OR | AOR^a^ |
|  | N=107 | N=31 | (95% CI) | (95% CI) |
| Clinical Stage |  |  |  |  |
| Stage I+ II | 49 (45.79) | 15 (48.39) | Reference | Reference |
| Stage III+IV | 58 (54.21) | 16 (51.61) | 0.90 (0.41-2.01) | 0.97 (0.41-2.10) |
|  |  |  |  |  |
| Tumor Size |  |  |  |  |
| T1 + T2 | 60 (56.07) | 23 (74.19) | Reference | Reference |
| T3 + T4 | 47 (43.93) | 8 (25.81) | 0.44 (0.18-1.08) | 0.44 (0.17-1.10) |
|  |  |  |  |  |
| Lymph node metastasis |  |  |  |  |
| Negative | 71 (66.36) | 19 (61.29) | Reference | Reference |
| Positive | 36 (33.64) | 12 (38.71) | 1.25 (0.55-2.85) | 1.23 (0.52-2.88) |
|  |  |  |  |  |
| Cell differentiation |  |  |  |  |
| Well differentiated | 15 (14.02) | 4 (12.90) | Reference | Reference |
| Moderately or poorly differentiated | 92 (85.98) | 27 (87.10) | 1.10 (0.34-3.60) | 1.18 (0.35-3.97) |

a. AOR adjusted, age, smoking status, alcohol intake and betel nut chewing.
