# Supplementary material for: Impacts of MicroRNA Gene Polymorphisms on the Susceptibility of Environmental Factors Leading to Carcinogenesis in Oral Cancer
Source: PLoS One. 2012 Jun 28;7(6):e39777. doi: 10.1371/journal.pone.0039777 (PMC3386241; doi:10.1371/journal.pone.0039777)
Supplement: Table S5 — Primer sequences and PCR conditions for amplification of miRNA SNPs. (DOC) [file pone.0039777.s005.doc]

Table S5. Primer sequences and PCR conditions for amplification of miRNA SNPs.

| Gene name | PCR primers | Enzyme | PCR product |
| --- | --- | --- | --- |
| miRNA146a rs2910164 | 5’-ATGGGTTGTGTCAGTGTCAGAGCT-3’  5’-TGCCTTCTGTCTCCAGTCTTCCAA-3’ | *Sac*I | C:122bp + 25bp  G:147bp |
| miRNA196  rs1161491 | 5’- CCCCTTCCCTTCTCCTCCAGATA-3’  5’- CGAAAACCGACTGATGTAACTCCG-3’ | *Msp*I | C:125bp + 25bp  T:149bp |
| miRNA499 rs3746444 | 5’- CAAAGTCTTCACTTCCCTGCCA-3’  5’- GATGTTTAACTCCTCTCCACGTGATC-3’ | *Bcl*I | T:120bp + 26bp  C:146bp |
